# In Vivo Cellular‐Level 3D Imaging of Peripheral Nerves Using a Dual‐Focusing Technique for Intra‐Neural Interface Implantation

**DOI:** 10.1002/advs.202102876

**Published:** 2021-11-29

**Authors:** Min Woo Lee, Namseon Jang, Nara Choi, Sungwook Yang, Jinwoo Jeong, Hyeong Soo Nam, Sang‐Rok Oh, Keehoon Kim, Donghyun Hwang

**Affiliations:** ^1^ Center for Intelligent and Interactive Robotics Korea Institute of Science and Technology Seoul 02792 Republic of Korea; ^2^ Department of Mechanical Engineering Korea Advanced Institute of Science and Technology Daejeon 34141 Republic of Korea; ^3^ Department of Mechanical Engineering Pohang University of Science and Technology Gyeongbuk 37673 Republic of Korea

**Keywords:** extending depth of focus, intra‐neural interface, neuroprosthesis, optical coherence tomography, peripheral nerve

## Abstract

In vivo volumetric imaging of the microstructural changes of peripheral nerves with an inserted electrode could be key for solving the chronic implantation failure of an intra‐neural interface necessary to provide amputated patients with natural motion and sensation. Thus far, no imaging devices can provide a cellular‐level three‐dimensional (3D) structural images of a peripheral nerve in vivo. In this study, an optical coherence tomography‐based peripheral nerve imaging platform that employs a newly proposed depth of focus extension technique is reported. A point spread function with the finest transverse resolution of 1.27 µm enables the cellular‐level volumetric visualization of the metal wire and microstructural changes in a rat sciatic nerve with the metal wire inserted in vivo. Further, the feasibility of applying the imaging platform to large animals for a preclinical study is confirmed through in vivo rabbit sciatic nerve imaging. It is expected that new possibilities for the successful chronic implantation of an intra‐neural interface will open up by providing the 3D microstructural changes of nerves around the inserted electrode.

## Graphical abstract

Optical coherence tomography‐based peripheral nerve imaging platform with a new depth of focus extension technique is reported. The microstructures of a rat sciatic nerve with a metal wire inserted are volumetrically visualized in vivo. It is expected that new possibilities for solving the chronic implantation problem of an intra‐neural interface will open up by providing the microstructural image of nerves.

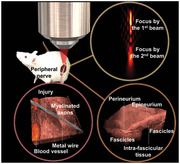

## 1 Introduction

Patients with amputations may desire prostheses that can perform the same function as their lost limb. Currently, the leading technology that satisfies this requirement is neuroprosthesis, wherein efferent motion intention is recorded to operate the prosthesis and afferent sensory percepts are elicited to enable the patient to feel sensations perceived in the prosthesis using neural interfaces.^[^ ^1^, ^2^ ^]^ Recent neuroprothesis studies employ an intra‐neural interface that directly inserts an electrode into the peripheral nerve; it demonstrates the possibility of implementing a high degree of freedom motion^[^ ^3^, ^4^ ^]^ or realizing natural sensation^[^ ^1^ ^]^ by exploiting the high selectivity of an intra‐neural interface. These interfaces were successful for most short‐term studies (less than 1 year) because of their high invasiveness.^[^ ^5^, ^6^ ^]^

Signal quality degrades when intra‐neural interfaces are used for more than several months, and this results in the problem of inferior functionality. Unfortunately, signal quality degradation is not easily solved because there are only few studies on the effects of the long‐term use of intra‐neural interfaces on signal quality degradation, and most of them rely on histological examinations of excised nerves.^[^ ^7^, ^8^ ^]^ However, microscopic anatomy of peripheral nerve fibers from the histological examinations only reflects its state at a specific instant. Further, it is difficult to make a histology slide that includes the electrode of the intra‐neural interface. Histology slides that are tens of micrometers thick are unsuitable for providing full volume information on the order of a few cubic millimeters. Therefore, extensive time and effort are required to obtain the histological information of the full volume of the peripheral nerve.^[^ ^9^, ^10^ ^]^

If an imaging device can obtain cellular‐level volumetric structural images of the peripheral nerve of the living animal with a fast imaging speed, it can help identify and solve cause of long‐term use problems of the inter‐neural interface. Unfortunately, such an imaging device has not been reported so far because peripheral nerve imaging is considerably challenging given the small dimensions and the heterogeneity. Magnetic resonance imaging and ultrasonography, used in clinical practice, can help visualize the peripheral nerve structure in vivo; however, their spatial resolution of hundreds of micrometers is insufficient to visualize cellular structures such as myelinated axons with a diameter of 12–20 µm.^[^ ^11^, ^12^, ^13^ ^]^ Recently, spectral confocal reflectance microscopy^[^ ^14^ ^]^ and coherent anti‐Stokes Raman spectroscopy^[^ ^15^ ^]^ were successfully applied to visualize the myelinated axons of rodent and rabbit sciatic nerves, respectively, in vivo. However, their narrow field of view (FOV) and nonvolumetric imaging properties provide information limited only to the nerve surface.

Optical coherence tomography (OCT) is a widely used three‐dimensional (3D) optical imaging technique in the biomedical field.^[^ ^16^, ^17^, ^18^, ^19^, ^20^ ^]^ In the late 1990s, researchers attempted in vitro imaging of the human peripheral nerves using OCT.^[^ ^21^, ^22^ ^]^ In the cross‐sectional OCT image of the peripheral nerve, fascicles were differentiated, but individual myelinated fibers were not resolved owing to insufficient transverse resolution of 30 µm. In 2010, it was demonstrated that in vitro OCT signal irregularities can be used to monitor peripheral nerve injury^[^ ^23^ ^]^ and repair; however, the microstructure of the elements that comprise the peripheral nerve was not visualized. To quantitatively assess the elements comprising the peripheral nerve, morphological features were extracted from the OCT with a transverse resolution of ≈20 µm.^[^ ^24^, ^25^ ^]^ However, fine structures such as myelinated axons could not be assessed. There have been attempts to visualize myelination using polarization sensitive OCT.^[^ ^26^, ^27^ ^]^ Although Nam et al. quantified the degree of myelination using vectorial birefringence imaging of the peripheral nerve, individual myelinated axons cannot be imaged because of limited spatial resolution.^[^ ^27^ ^]^ Arous et al. visualized the myelinated axons of the rat sciatic nerve using full‐field optical coherence microscopy, with a transverse resolution of 2.2 µm.^[^ ^28^ ^]^ However, because of the depth scan requirement, acquiring a volumetric image requires considerable time. Recent attempts to visualize the human peripheral nerve using commercially available OCT have been unsuccessful in clearly distinguishing myelinated axons because of the motion artifacts, which are generated due to the slow imaging speed.^[^ ^13^, ^29^ ^]^ To increase the imaging speed, several techniques have been developed, such as stretched‐pulse mode‐locking based circular‐ranging OCT,^[^ ^30^, ^32^ ^]^ Fourier‐domain mode‐locking laser technique,^[^ ^33^, ^34^, ^35^ ^]^ and space‐division multiplexing OCT.^[^ ^36^, ^37^, ^38^ ^]^ These techniques enable A‐line rate of several MHz to near 1 MHz. However, the spectral ranges of the laser sources, with an axial resolution of less than 5 µm, are unsuitable for ultrahigh‐resolution imaging.

In this study, we demonstrate an OCT‐based peripheral nerve imaging platform that overcomes the limitations of currently available imaging techniques. We applied a newly proposed dual‐focusing technique that extends the depth of focus (DOF), which enables the acquisition of a high‐resolution volumetric image without additional depth scanning. Initially, the in vivo volumetric imaging of a rat sciatic nerve was performed to confirm the ability to visualize microstructures; their morphological features in OCT images were compared with the histology images. Next, the volumetric visualization of nerve damage was confirmed via the in vivo imaging of rat sciatic nerve inserted with an electrode mimicking nickel‐titanium (Ni‐Ti) wire. Finally, we demonstrate the feasibility of utilizing our imaging platform for large animals through the in vivo imaging of the rabbit sciatic nerve, which has a nerve size like that of humans.

## 2 Results

### 2.1 Peripheral Nerve Imaging Platform Based on Custom‐Built

**Figure 1:**
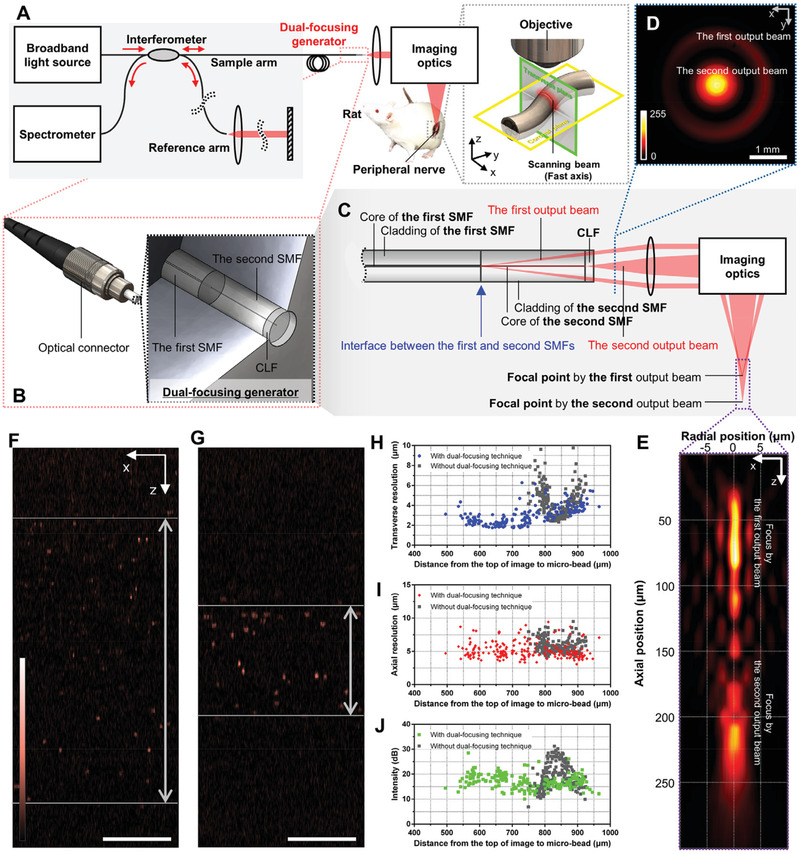
Optical coherence tomography‐based peripheral nerve imaging platform including a dual‐focusing generator. A) Simplified schematic of peripheral nerve imaging platform and definition of anatomical plane. Broadband light is guided to the sample and reference arms through the fiber‐optic coupler. Backscattered interference signals of the sample and reference arms are detected using a spectrometer. Black solid lines: optical fibers. B) Schematic of dual‐focusing generator located inside an optical connector. C) Principle of DOF extension using the dual‐focusing generator. Two beams divided at the interface between the single‐mode fiber (SMF)s are focused on two different axial positions. SMF, single‐mode fiber; CLF, coreless fiber. D) Measured radial intensity profiles of output beams from the distal end of the dual‐focusing generator; two distinct beams were observed. E) Measured transverse point spread function. The two focal points generated by the two output beams extend the entire DOF. Cross‐sectional OCT images of microbeads F) with and G) without the dual‐focusing generator. The scale bar: 100 µm. H) A plot of transverse resolution and distance from the top of the image to the microbead. I) A plot of axial resolution and distance from the top of the image to the microbead. J) A plot of intensity and distance from the top of the image to the microbead.

We developed a peripheral nerve imaging platform based on a custom‐built OCT (**Figure** **1**A and Figure S1A, Supporting Information) for visualizing cellular‐level volumetric microstructures of peripheral nerves with high imaging speed in vivo. A broadband light source allows an axial resolution of 4.5 µm (Figure S1B, Supporting Information), which enables the discrimination of individual myelinated axons. Guided light through the sample arm is raster scanned by the imaging optics. A single raster scanned area is 1.00 × 1.00 mm^2^ (x, y, z in Figure 1A), and it includes representative elements that comprise the peripheral nerve such as myelinated axons, connective tissues, and blood vessels. This large volume can help understand a structural change in the nerve caused by electrodes that enable the simultaneous observation of interactions among elements that make up the nerve. The A‐line rate of 250 kHz enables to acquire a single volumetric image within 2.56 s with pitch of 1.56 µm (640 frames within 1.0 mm).

### 2.2 DOF Extending Performance of Dual‐Focusing Generator

Unlike other peripheral nerve imaging devices, our imaging platform employs a dual‐focusing technique to extend the DOF; this allows cellular‐level volumetric imaging without additional depth scanning. The newly proposed DOF extending technique is implemented in the dual‐focusing generator of the sample arm in the form of an optical connector for protection, maintenance, and stable alignment (Figure 1B). The dual‐focusing generator comprises two single‐mode fibers (SMFs) and a coreless fiber (CLF). The dual‐focusing generating part includes two SMFs; since SMFs have different core diameters and refractive indices, the output beam is divided into two beams near the interface between the first and second SMF (Figure 1C). The first and second output beams are guided through the cladding and core of the second SMF, respectively. The two output beams of the dual‐focusing generator are confirmed by the imaging output beams using a beam profiler (Figure 1D and Figure S2A, Supporting Information). Since the first and second output beams arise from different axial positions, they focus on different axial positions after the imaging optics. Thus, two focal points at different axial positions extend the entire DOF; this was confirmed by measuring the point spread function (PSF) (Figure 1E, Figures S2B and S3–S5, Supporting Information). The finest transverse resolution was 1.27 µm, and this is sufficient for cellular‐level imaging. The entire extended DOF was 240 µm, which is 5.1 times longer than that of the Gaussian beam propagation. In order to compare the DOF extending tendencies in the OCT image with or without the dual‐focusing generator, we performed microbead imaging and analysis. In the cross‐sectional OCT images of the microbead, the range in which the microbeads are in focus is wider when applying the dual‐focusing generator (Figure 1F) compared to that without one (Figure 1G). The variations in transverse resolutions as a function of the depth show an extended DOF (Figure 1H) at a glance. Axial resolutions have similar values in the entire DOF (Figure 1I) with and without the dual‐focusing generator; this means our dual‐focusing generator does not truncate the source spectral band. A sensitivity loss is observed on a plot of intensity and distance from the top of the image to the microbead (Figure 1J). The sensitivities, measured using mirror and neutral density filter having optical density of 2 with and without the dual‐focusing generator were 86.54 dB and 94.41 dB, respectively. Despite the sensitivity loss, extending the DOF is valuable for imaging the peripheral nerve without additional depth scanning. The combination of no additional depth scan by the extended DOF and the A‐line rate of 250 kHz allowed significantly suppress motion artifacts caused by breathing. (Supporting information and Figure S6, Supporting Information).

### 2.3 In Vivo Imaging of Myelinated Axons

**Figure 2:**
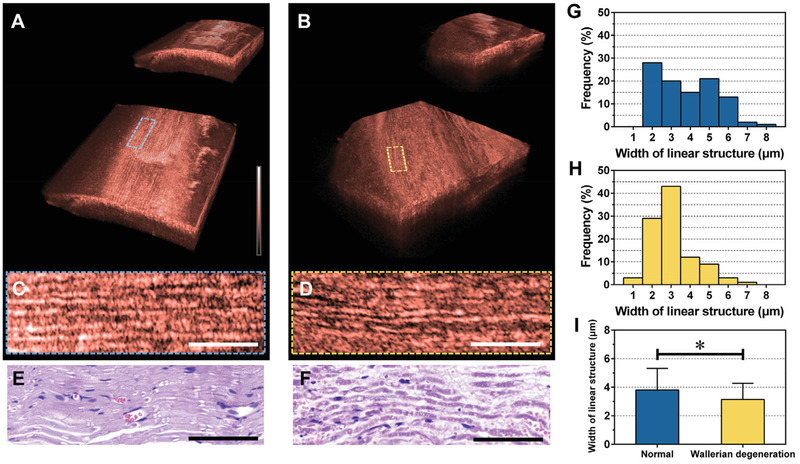
In vivo microstructure images of myelinated axons in rat sciatic nerve. 3D rendering images of A) normal sciatic nerve and B) sciatic nerve with Wallerian degeneration (WD). Magnified coronal plane images of C) normal myelinated axons (NM) and D) myelinated axons with WD. Hematoxylin and eosin (H&E) stained tissue slide images of E) NM and F) WD. Histogram of the width of the linear structure in G) NM and H) WD. I) Comparison of the averaged width between NM and WD. Measured values are presented as means ± standard deviation. An unpaired *t*‐test was performed with *n* = 100. *P < 0.0006 denotes significant difference. All scale bars: 100 µm.

We acquired cross‐sectional OCT images of myelinated axons of the rat sciatic nerve in vivo. Since the myelinated axons play an essential role in neural signal conduction in vertebrates,^[^ ^39^ ^]^ the structural changes of myelinated axons caused by the insertion of the electrode can be considered a major cause for signal quality degradation during the long‐term implantation of the intra‐neural interface. We compared the OCT images of normal myelinated axons (NM) and axons in which myelin sheaths were destroyed because of Wallerian degeneration (WD),^[^ ^40^ ^]^ one of the representative processes in which structural changes of myelinated axons occur. The 3D rendering images of the normal sciatic and sciatic nerves with WD are shown in **Figure** **2**A,B, respectively. We identify the exterior and interior volumetric microstructures of the nerve in the full‐ (upper‐right images in Figure 2A,B) and cut‐view (large images in Figure 2A,B) 3D images, respectively. The representative magnified coronal sections of NM and axons with WD are shown in Figure 2C,D, respectively; they correspond to the region indicated with the blue and yellow dashed boxes in Figure 2A,B. In the coronal section of the NM, the array of the linear structures of axons was observed clearly. Broken linear structures were observed in the cross‐sectional image of axons with WD. Further, the width of the linear structure with WD appeared to be irregular, and it was smaller than that of NM caused by the destruction of myelination, which is a typical pathological phenomenon of WD.^[^ ^41^ ^]^ The fine linear structure of the NM and broken structure of the axon with the WD of OCT images are identified in hematoxylin and eosin (H&E) stained tissue slide images that show the general morphological features of the peripheral nerve (Figure 2E,F).^[^ ^42^ ^]^

For quantitative analysis (Figure S7, Supporting Information), the widths of the linear structures were measured and presented as plots of a histogram. The histograms of normal (Figure 2G) and WD (Figure 2H) confirm that axons with the WD had smaller widths. The difference in the width between the NM and axons with the WD was statistically significant (3.80 ± 0.15 µm versus 3.14 ± 0.11 µm, *p* < 0.0006, Figure 2I). Therefore, our imaging platform can visualize individual myelinated axons and distinguish injured myelinated axons from NM.

### 2.4 In Vivo Imaging of Connective Tissues

**Figure 3:**
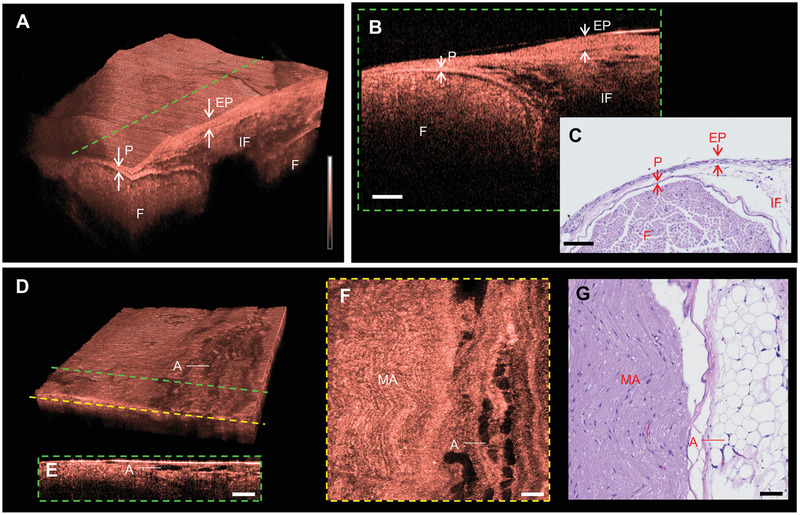
In vivo microstructure images of connective tissues in the rat sciatic nerve. A) 3D rendering image of the rat sciatic nerve including the epineurium and perineurium. B) Representative transverse plane image of epineurium and perineurium corresponding to the cross‐section by green dashed line in A. C) H&E stained tissue slide images of the epineurium and perineurium. D) 3D rendering image of the rat sciatic nerve including the adipose tissue. Representative E) transverse plane and F) coronal plane images of adipose tissue corresponding to the cross‐section indicated by the green and yellow dashed line in D. G) H&E stained tissue slide images of adipose tissue. All scale bars: 100 µm. EP, epineurium; P, perineurium; F, fascicle; IF, intra‐fascicular tissue; A, adipose tissue; and MA, myelinated axons.

We used the imaging platform to visualize the connective tissues of the rat sciatic nerve, such as the epineurium, perineurium, and adipose tissue in vivo. The epineurium—the outermost layer of the peripheral nerve—protects the interior elements of the nerve. While implanting the intra‐neural interface, injury to the epineurium is unavoidable; the injured epineurium can cause epineural fibrosis. Epineural fibrosis can reduce nerve flexibility, which makes the nerves vulnerable to pressure, and in severe cases, it can lead to ischemia, which can interrupt the function of the intra‐neural interface. Since epineural fibrosis appears as a change in the thickness of the epineurium,^[^ ^43^ ^]^ the accurate measurement of the epineurium thickness can help in its identification. We confirmed whether the epineurium images obtained by our imaging platform can be used to measure the thickness of the epineurium. As shown in the 3D rendering image (**Figure** **3**A), the epineurium is observed as a distinguishable thick membrane that surrounds fascicles and interfascicular tissues. In a transverse plane (Figure 1A) image (Figure 3B) corresponding to the cross‐section represented by a green dashed line, the epineurium appears as a distinguished thick band. As can be seen intuitively from the images, the thickness of the epineurium can be accurately measured without any post image processing because of the high spatial resolution. Even in the H&E stained tissue slide image, the epineurium is observed as a thick band shape similar to that of the OCT image. This finding suggests that our imaging platform is useful for identifying epineural fibrosis caused by the implantation of the intra‐neural interface in vivo.

The perineurium, which directly surrounds the fascicle, plays an important role in regulating the pressure inside the fascicle by blocking the inflow of the epineural fluid.^[^ ^44^ ^]^ Since the perineurium is inevitably injured when implanting the intra‐neural interface, it can be necessary to check the healing process of the perineurium. However, imaging devices that can visualize the perineurium in vivo are scarce because of their thickness of only ≈4 µm.^[^ ^45^ ^]^ Nevertheless, owing to the superior spatial resolution of our imaging platform, the perineurium is clearly differentiated from other elements as a highly scattered thin membrane and band in the 3D image (Figure 3A) and transverse plane image (Figure 3B), respectively. The thin band structure of the perineurium is observed in the histology image (Figure 3C). The volumetric OCT image of the perineurium revealed the capability of the imaging platform to identify whether the perineurium is ruptured by the implantation of the intra‐neural interface.

Adipose tissue is distributed around and within the nerve to protect the nerve from compression by absorbing pressure. We imaged the adipose tissue of the rat sciatic nerve in vivo using our imaging platform. A 3D cut‐view rendering image of the adipose tissue is illustrated in Figure 3D. The shape of the cumulus, which is a typical morphological feature of the adipose tissue, is clearly observed in the 3D image. The honeycomb structure of the adipose tissue was observed in the transverse (Figure 3E) and coronal (Figure 3F) plan images corresponding to the cross‐section indicated by the green and yellow dashed lines in Figure 3D. This feature is well matched with that of the histology image (Figure 3G).

### 2.5 In Vivo Imaging of Blood Vessels

**Figure 4:**
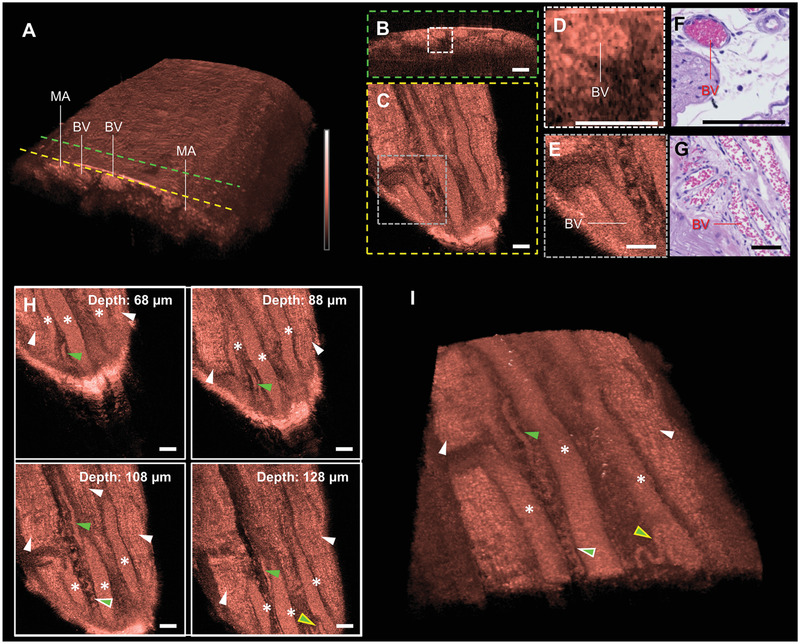
In vivo microstructure images of blood vessels in rat sciatic nerve. A) 3D rendering image of sciatic nerve including blood vessels. Representative B) transverse and C) coronal plane images of blood vessels corresponding to cross‐section indicated by green and yellow dashed lines, respectively in (A). Magnified D) transverse and E) coronal plane images of blood vessels corresponding to the white and gray dashed box in (B) and (C), respectively. H&E stained tissue slide images of blood vessels in F) transverse and G) coronal planes. MA, Myelinated axons; BV, blood vessel. H) Sequential coronal plane images of the rat sciatic nerve based on the depth of myelinated axons (white arrow heads), large blood vessels (white asterisks), and small blood vessels (green arrow heads). I) Corresponding 3D rendering image of rat sciatic nerve with epineurium removed manually. All scale bars: 100 µm.

Blood vessels play an essential role in nerve survival by supplying nutrients to the nerves.^[^ ^46^ ^]^ Thus, repeated blood vessel rupture by the motion of electrode can be considered a possible cause that makes the chronic implantation of the intra‐neural interface difficult. We confirmed if our imaging platform can visualize blood vessels by imaging a rat sciatic nerve in vivo. **Figure** **4**A shows the 3D volume rendering image of the rat sciatic nerve. The cross‐sections of blood vessels and myelinated axons are shown in the figure. Owing to the strong scattering of red blood cells, the blood vessels appeared as highly scattered round shapes and branched structures in the transverse (Figure 4B) and coronal (Figure 4C) plane images that correspond to the cross‐section indicated by the green and yellow dashed lines, respectively, in Figure 4A. These morphological features were confirmed in both magified OCT images (Figure 4D,E) in the histology image (Figure 4F,G). The microstructure of the blood vessels can be clearly understood from sequential coronal plane images in the depth direction. Figure 4H shows the microstructure of blood vessels and myelinated axons at specific depths. By observing the coronal plane images at sequential depths, the location and shape of large (white asterisks) and small blood vessels (green arrow heads) such as vasa nervorum around the wave structures of myelinated axons (white arrow heads) can be identified clearly. The spring (green arrow heads with white border) and hairpin (green arrow heads with yellow border) structures of small blood vessels can be identified by viewing the 3D volume rendering image (Figure 4I and Video S1, Supporting Information) that is difficult to grasp in a histology image where only a specific cross‐sectional structure can be observed. These findings imply that our imaging platform can be used to check blood vessel rupture caused by the motion of the implanted intra‐neural interface.

### 2.6 Imaging of Sciatic Nerve with Nickel‐Titanium Wire Inserted Immediately after Euthanasia

**Figure 5:**
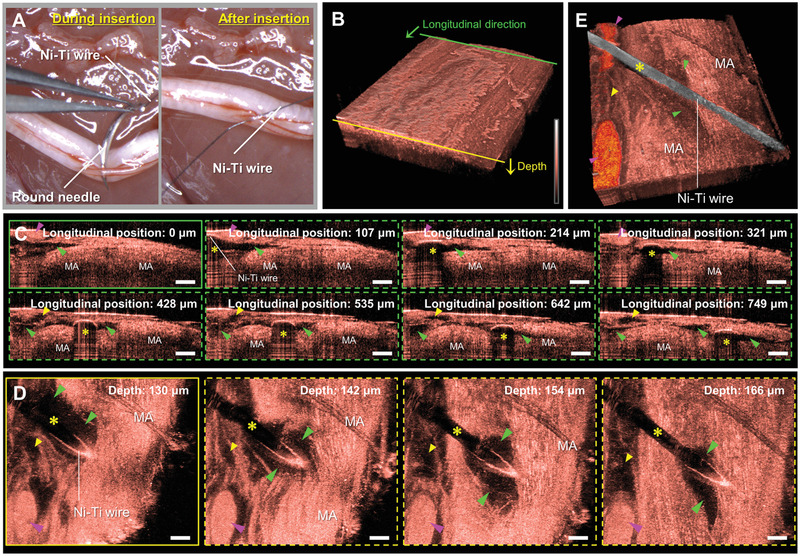
Microstructural changes of rat sciatic nerve with nickel‐titanium (Ni‐Ti) wire inserted immediately after euthanasia. A) Photographs of rat sciatic nerve during and after a Ni‐Ti wire insertion. B) 3D rendering image of the sciatic nerve, obtained immediately after euthanasia. C) Sequential transverse plane images of the sciatic nerve with the dark shadow of the Ni‐Ti wire (yellow asterisks), blood vessels (magenta arrow heads), injured blood vessels (yellow arrow heads), and injured tissues (green arrow heads) according to the longitudinal position from the cross‐section indicated by the solid green line in (B). D) Sequential coronal plane images of the sciatic nerve with same morphological features as those of (C) according to the depth from the cross‐section indicated by the solid yellow line in (B). E) Color‐coded 3D rendering images of the sciatic nerve with the epineurium removed manually. All scale bars: 100 µm. MA, myelinated axons.

We confirm if our imaging platform can be used to discern the electrode‐induced structural changes in the peripheral nerve, the rat sciatic nerves damaged by the insertion of Ni‐Ti wire (**Figure** **5**A) were imaged immediately after euthanasia. To mimic the electrode, a Ni‐Ti wire with a diameter of 50 µm, similar in size to the Utah slanted electrode array—representative electrode for the intra‐neural interface—was used. In the 3D rendering image (Figure 5B) of the rat sciatic nerve with the Ni‐Ti wire inserted, myelinated axons, blood vessels, and epineurium were observed volumetrically. Figure 5C represents the sequential transverse plane images based on the longitudinal position from the cross‐section indicated by the solid green line in Figure 5B. In the cross‐sections, the Ni‐Ti wire appeared as a highly reflected round surface with dark shadows (yellow asterisks). The blood vessels were seen as strongly scattered ellipses (magenta arrow heads). The injured blood vessel (yellow arrow heads) and tissues (green arrow heads) were viewed as dark cavities. It is evident that the round needle and Ni‐Ti wire tear and enter the nerve (Video S2, Supporting Information). Figure 5D shows the sequential coronal plane images of the sciatic nerve according to the depth from the cross‐section indicated by the solid yellow line in Figure 5B. Interestingly, in the coronal plane, the strands of the torn myelinated axons are observed near the Ni‐Ti wire. In addition, cavities that are considerably wider than the size of the Ni‐Ti wire around the Ni‐Ti wire indicate the trace of the large round needle because the dimension of the cavity is consistent with the width of the round needle of 0.56 mm. These morphological features are represented as color‐coded 3D rendering images with the epineurium removed manually (Figure 5E and Video S3, Supporting Information). Thus, it was confirmed that our imaging platform is a useful tool for examining the microstructural changes of nerves with the intra‐neural interface implanted.

### 2.7 Imaging of Sciatic Nerve with Nickel‐Titanium Wire Inserted In Vivo

**Figure 6:**
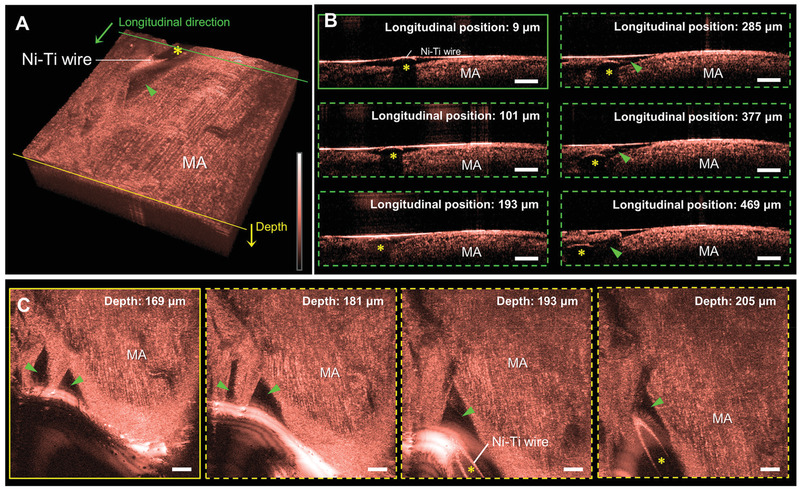
In vivo microstructural changes of the rat sciatic nerve with the nickel‐titanium (Ni‐Ti) wire or electrode inserted and ex vivo microstructure images of myelinated axons in the rabbit sciatic nerve. A) 3D rendering image of the rat sciatic nerve with Ni‐Ti wire inserted, obtained in vivo. B) Sequential transverse plane images of the sciatic nerve with dark shadow of the Ni‐Ti wire (yellow asterisks), and injured tissues (green arrow heads) according to the longitudinal position from the cross‐section indicated by the solid green line in (A). C) Sequential coronal plane images of the sciatic nerve with the same morphological features as those of (B) according to the depth from the cross‐section indicated by the solid yellow line in (A). D) 3D rendering images of the rabbit sciatic nerve, obtained ex vivo. E) Magnified coronal plane image of myelinated axons corresponding to the yellow dashed box in (D). All scale bars: 100 µm. MA, myelinated axons.

The rat sciatic nerve with the Ni‐Ti wire inserted was imaged using our imaging platform in vivo to demonstrate the capability of imaging the peripheral nerve with the Ni‐Ti wire inserted in vivo. In the 3D rendering image (**Figure** **6**A and Video S4, Supporting Information), similar structural features to those of imaging immediately after euthanizing the rat, such as highly reflected round surface and dark shadow (yellow asterisks) of the Ni‐Ti wire and the cavity of injured tissues (green arrow heads) were visualized. Fine line structures of the myelinated axons were also observed. Similar to results of imaging immediately after euthanizing the rat, The Ni‐Ti wire, cavity of injured tissues, and myelinated axons were also clearly observed in sequential transverse (Figure 6B) and coronal (Figure 6C) plane images. However, when imaging the rat sciatic nerve with the Ni‐Ti wire inserted, the sensitivity of the OCT images appears to be slightly lower than that immediately after the euthanasia imaging results. The cause of the low sensitivity issue is attributed to the strong absorption by blood, which occurs during the insertion of the Ni‐Ti wire. If a blood suction device can be introduced, even during the electrode insertion or immediately after electrode insertion, our imaging platform can reveal microstructural changes of the nerve with adequate sensitivity.

### 2.8 In Vivo Imaging Rabbit Sciatic Nerve

**Figure 7:**
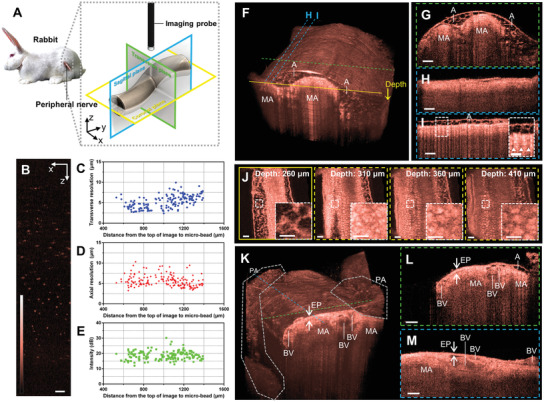
In vivo rabbit sciatic nerve imaging. A) Definition of anatomical plane. B) Cross‐sectional OCT image of microbeads. As function of the distance from the top of the image to the microbead, a plot of C) transverse resolution, D) axial resolution, and E) intensity. F) 3D rendering image of rabbit sciatic nerve. G) Transverse sections of rabbit sciatic nerve corresponding to cross‐section represented by the green dashed line in (F). H, I) Sagittal plane images of rabbit sciatic nerve corresponding to the cross‐section indicated by the blue dashed lines in (F). J) Sequential coronal sections of rabbit sciatic nerve corresponding to the cross‐section represented by the yellow solid line in (F). K) 3D rendering image of adipose tissue peeled rabbit sciatic nerve. L) Transverse and M) Sagittal sections of adipose tissue peeled rabbit sciatic nerve corresponding to cross‐sections indicated by the green and blue dashed lines in (K), respectively. All scale bars are 100 µm except for the magnified images (white dashed box) for which the sale bar is 50 µm. MA, myelinated axons; A, adipose tissue; PA, peeled adipose tissue; EP, epineurium; and BV, blood vessel.

We imaged the rabbit sciatic nerve for which the myelinated axon diameter is similar to that of the human myelinated axon^[^ ^13^ ^]^ in vivo to confirm the feasibility of applying our imaging platform for preclinical studies using a large animal (**Figure** **7**A). Since the rabbit sciatic nerve is located deep in the thigh, we changed the imaging optics to the custom‐designed gradient‐index (GRIN) lens‐based imaging optics for which the diameter was only 2.7 mm. Further, we modified the imaging performance to increase the transverse resolution and DOF caused by the larger myelinated axons and sciatic nerve of the rabbit. A microbead solution was imaged and analyzed to confirm the modified imaging performance (Figure 7B). The transverse resolution below 7.5 µm ranged over ≈750 µm of depth (Figure 7C). Axial resolutions in a similar depth range have values like that of the imaging optics used for the rat imaging; this implies the modified imaging optics did not truncate the light spectrum (Figure 7D). In addition, the intensity distribution was uniform in the entire DOF (Figure 7E).

Figure 7F shows the 3D rendering image of the rabbit sciatic nerve. In the transverse section (Figure 7G), the myelinated axons and adipose tissue are observed as bright spots and honeycomb structures. Further, in the sagittal section (Figure 7H), the myelinated axons show a linear structure similar to that of rat imaging. However, there is adipose tissue that surrounds the nerve (Figure 7I); the linear structures of the myelinated axons are not clearly distinguished because of the strong shadowing caused by the absorption of the adipose tissue (white arrow heads). The shadowing leads to artifacts in the coronal sections. Although there is no adipose tissue, the dark honeycomb structures similar to those of outer adipose (Figure 7J, depth: 260 µm) are observed to be deeper than 400 µm. Therefore, the outer adipose tissue should be peeled to avoid shadowing artifacts.

The 3D rendering images of adipose tissue peeled rabbit sciatic nerve is shown in Figure 7K and Video S5, Supporting Information. The remaining adipose tissue surrounds the nerve in the shape of the cumulus (white dashed polygon). In both the transverse (Figure 7L) and sagittal (Figure 7M) sections, the epineurium and blood vessels were observed as a thick band and a round shape, respectively. Especially, the blood vessels were more clearly visible in the real‐time images because of the motion of red blood cells (Video S6, Supporting Information). These results demonstrate that our imaging platform can be used for imaging the peripheral nerve of a large animal.

## 3 Discussion

We presented an OCT‐based imaging platform that can provide the 3D structural features of a rat sciatic nerve with a cellular‐level resolution in vivo. Our novel DOF extending technique allows to obtain a single volumetric image within 2.56 s and visualize the cross‐sectional images in real‐time without additional depth scanning. Further, our imaging platform allows the volumetric visualization of the electrode mimicked Ni‐Ti wire and the structural changes inside the nerve in vivo. In addition, the feasibility of applying our imaging platform to the large animal was verified using the GRIN optics‐based imaging probe.

Since the trade‐off between the transverse resolution and DOF is a crucial problem for the ultra‐high resolution OCT, several extending DOF techniques such as apodization,^[^ ^47^ ^]^ axicon lens,^[^ ^48^ ^]^ and binary phase filter^[^ ^49^ ^]^ have been developed. Although these methods are representative strategies for extending DOF, they have certain limitations such as loss of light, difficulty of alignment, fabrication, and design. Using multimode waveguide^[^ ^50^, ^51^ ^]^ is recently developed method which is simple and easy to align and fabricate without loss of light. However, a multimode waveguide requires ≈1.2 mm of multimode fiber to generate sufficiently higher modes. Our dual‐focusing generator is not only easy to align and fabricate without loss of light but also requires only ≈0.4 mm of the SMF, which is an advantage where the rigid part needs to be as short as possible, such as miniaturization. There are other types of DOF extending techniques such as interferometric synthetic aperture microscopy^[^ ^52^ ^]^ and optical coherence refraction tomography.^[^ ^53^ ^]^ These techniques use multiple scanning to correct and reconstruct images with an extended DOF. Since these techniques use Gaussian beam, which is different from the previously described Bessel‐beam based beam shaping techniques, side lobe artifacts and sensitivity losses do not occur. However, post‐processing requirements deem them unsuitable for high‐speed volumetric in vivo imaging or real‐time visualization yet. Therefore, the dual‐focusing generator with no post‐processing requirement can be more suitable for in vivo imaging of the peripheral nerve microstructure.

The dual‐focusing generator causes side lobe artifact and sensitivity loss. The side lobe artifact is a general problem of the DOF extending technique using Bessel beam. By analyzing the PSF, we confirmed that the minimum difference between the central and side lobes is 5.53 dB (Figure S3D, Supporting Information) which is not low enough value to be eliminated through contrast control. In addition, considerable modulation caused by side lobe was observed in the simulation for multiple scattering (Figure S4, Supporting Information). Therefore, it can make the side lobe artifacts in the OCT images when imaging multiple scattering samples. However, most range of the PSF has a value higher than 5.53 dB, as shown in Figure S3C,E, Supporting Information. In addition, few significant side‐lobe artifacts were observed when OCT imaging was performed on a fresh graph (Figure S5, Supporting Information). Therefore, our dual‐focusing technique is still valuable for high‐speed or real‐time imaging of the microstructure of the peripheral nerve in spite of the side lobe artifact. In addition, sensitivity loss is an intrinsic property of the dual‐focusing generator. Main cause of the sensitivity loss is redistribution of the light energy because loss of throughput or back‐coupling in the dual‐focusing generator was not observed. With the dual‐focusing generator, the same amount of light energy is redistributed over a wider range compared to that without the dual‐focusing generator. Thus, the illuminated energy per scatterer becomes decreases leading to lower sensitivity. However, although a sensitivity loss of 7.87 dB between mirror measurements with and without the dual‐focusing generator is not a low value, it allows our imaging platform to focus the beam over a wider range without additional depth scanning.

In the current imaging setup that uses a 2‐axis galvanometer mirror, the FOV is kept narrow for the preclinical study using an electrode of several millimeters. The narrow FOV problem can be solved using both, a single‐axis galvanometer mirror and a translational stage caused by the thin and long morphological characteristics of the peripheral nerve. Nam et al. demonstrated a wide‐field imaging of a rat sciatic nerve using a single‐axis galvanometer mirror and a translational stage.^[^ ^27^ ^]^ However, because the nerve height is not constant, the height of the imaging optics is not adjusted, and the nerve may go out of the imaging range. Thus, the height variation needs to be compensated using the height profile measured in advance. Further, the narrow DOF of a high‐resolution OCT compels the tight positioning of imaging optics even if the extending DOF technique is applied. Therefore, the imaging platform is integrated with a microsurgical robotic system to compensate for the nerve height variation. The precision^[^ ^54^, ^55^ ^]^ and depth servoing^[^ ^56^ ^]^ of the robotic system allow us to maintain the nerve position in the imaging range. Additionally, in wide‐field imaging without high‐speed lasers, owing to the motion artifacts generated from breathing, the reconstructed 3D volume image is inevitably distorted.^[^ ^31^ ^]^ However, currently developed high‐speed lasers are unsuitable for high‐resolution imaging which requires a broadband spectral range. Therefore, motion artifact‐correcting algorithms can help reconstruct 3D volume image.^[^ ^57^ ^]^

Our imaging platform not only expands the histological examination‐based studies for determining the cause of signal degradation to study living peripheral nerve microstructure, but it can also be applied to preclinical studies utilizing primates wherein histological examinations cannot be performed. When severe signal quality degradation occurs in the primate study, an intra‐neural interface is removed and the experiment is discontinued without identifying the cause of signal degradation because there was no imaging tool for the microstructure of the peripheral nerve in vivo. Therefore, our imaging platform that can visualize the microstructure of the peripheral nerve in vivo can be useful for identifying the cause of signal degradation in a preclinical study.

Our imaging setup requires exposing the nerve through a surgical procedure for imaging the peripheral nerve. However, the repeated exposure of the nerve when serial imaging is required can cause severe inflammation. We expect the repeated exposure problem to be solved through the miniaturization of imaging optics and by applying the imaging channel and mounting to the limb with electrodes inserted similar to those used for brain imaging.^[^ ^58^, ^59^ ^]^

Our animal models have some limitations. To observe the structural changes in the myelinated axons, we used the WD model. Although WD is one of the representative processes in which the structural changes of myelinated axons occur, it cannot completely reflect the changes in acute injury induced by intra‐neural interface implantation. In addition, although our imaging platform can be used when thickness of the epineurium should be measured such as epineural fibrosis, our rat model was not an epineural fibrosis model. Thus, if the thickness of the epineurium is greater than the imaging range or if the refractive index of the epineurium comes closer to that of the perineurium, our imaging platform would be of limited use. Finally, the number of animals for imaging was not enough to analyze statistical significance but adequate for a proof‐of‐concept. To overcome these limitations, a subsequent study will be conducted in primates of which conditions are most similar to those of clinical study. We plan to study for investigating the cause of signal quality degradation for structural changes such as changes in nerve fiber diameter and, epineurium thickness, and encapsulation of the electrode in the intra‐neural interface inserted to the peripheral nerve of the primate.

## 4 Conclusion

In conclusion, our imaging platform is a powerful tool for studies investigating the effect of the chronic implantation of intra‐neural interfaces on signal quality degradation and for guiding the successful implantation of the intra‐neural interface. Such studies can be considered as the basis for the development of neural interfaces that can provide a high degree of freedom of movement and natural sensations to patients with amputations. We expect the development of this imaging platform to be the beginning of research that will change the paradigm of the current neuroprosthetic field.

## 5 Experimental Section

### Peripheral Nerve Imaging Platform Based on Custom‐Built OCT

A spectral‐domain OCT‐based imaging platform was developed for visualizing microstructures of the peripheral nerve (Figure S1A, Supporting Information). The broadband light source (EXW‐4 OCT, NKT Photonics A/S, Birkerød, Denmark, in Figure S1A(1) and C(1), Supporting Information) was filtered using a short‐pass filter F1 (FF01‐940/SP‐25, Semrock, NY, USA) and a long‐pass filter F2 (FF01‐776/LP‐25, Semrock, NY, USA). Thus, light with a spectral range of 780–900 nm was coupled to a fiber‐optic coupler (TW850R5A2, THORLABS, NJ, USA, in Figure S1A(2) and C(2), Supporting Information) using a plano‐convex lens L1 (*f* = 7.5 mm, 67460, Edmund Optics, NJ, USA) to flatten the spectrum. In the sample arm (in Figure S1A(3) and C(3), Supporting Information), the output beam from the dual‐focusing generator was collimated using a collimating lens L2 (*f* = 7.5 mm, AC050‐008‐B, THORLABS, NJ, USA) and expanded using achromatic lenses L3 (AC254‐030‐B, THORLABS, NJ, USA) and L4 (AC254‐050‐B, THORLABS, NJ, USA). The expanded beam was focused on the sample using objective lens L5 (CFI Plan Fluor, 20×, Nikon, Tokyo, Japan). Raster beam scanning was performed using a two‐axis galvanometer mirror (GVS002, THORLABS, NJ, USA). The fast scan axis was for the *x*–axis, as shown in Figure 1A. Optics with similar dispersion as used for the sample arm were used for the reference arm (in Figure S1A(4) and C(4), Supporting Information). The interference signal was detected using a spectrometer (CS800‐840/120‐250‐OC2K‐CL, Wasatch Photonics, NC, USA, in Figure S1A(5) and C(5), Supporting Information) and digitized using a frame grabber (Xtium‐CL MX4, Teledyne DALSA, Ontario, Canada). The trigger signal synchronized with data acquisition was generated on a sync board (NI USB‐6343, National Instruments, TX, USA, in Figure S1A(6) and C(6), Supporting Information) and transferred to the galvanometer mirror through its driver. The volumetric image size including total axial range was 1.0 × 1.0 × 2.1 mm^3^ (x, y, z in Figure 1A) comprising 918 × 640 × 1010 voxels.

When the rabbit sciatic nerve was imaged in vivo, the imaging optics were modified to increase the transverse resolution and DOF because the rabbit sciatic nerve and its myelinated axons were larger than those of a rat. The achromatic lenses L3 and L4 were changed to achromatic lenses with an effective focal length of 50 and 35 mm, respectively, to reduce the beam size. An objective lens L5 was changed to combine two GRIN lenses with diameters of 2.7 mm for easy access to the sciatic nerve located deep in the thigh. The first GRIN lens (SRL‐270‐050‐2‐0‐0‐3, Go!Foton, NJ, USA) with a low gradient constant was used as the relay lens. The second GRIN lens (ILW‐2.70‐0.06, Go!Foton, NJ, USA) with a high gradient constant was used as the focusing lens.

A cross‐sectional image of a mirror near the position where the optical path difference between the sample and reference arms was zero was acquired using the imaging platform to measure the axial resolution. The linear scaled axial profile of the mirror was Gaussian fitted using MATLAB R2019a (MathWorks, MA, USA). The axial resolution was determined as the full width half‐maximum (FWHM) of Gaussian fitted data. The measured and Gaussian fitted data were presented after normalization using the peak value of the Gaussian fitted data (Figure S1B, Supporting Information).

### Fabrication of Dual‐Focusing Generator

A dual‐focusing generator was developed for extending the DOF. The dual‐focusing generator consisted of the first (780HP, Nufern, CT, USA) and second (SM300, FIBERCORE, Southampton, UK) SMFs and CLF (MM125, FIBERCORE, Southampton, UK). The optical fibers were spliced and cleaved using a fusion splicer (Swift S5, UCL SWIFT, Daejeon, Republic of Korea) and fiber cleaver (LDC400, THORLABS, NJ, USA), respectively. A ferrule connector/physical contact connector (1060533170, Molex, IL, USA) was used to protect the dual‐focusing generator. The distal end surface of the connector was polished using a hand polishing puck.

The beam output from the distal end of the dual‐focusing generator was imaged using a beam profiler (BC106N‐VIS/M, THORLABS, NJ, USA) to confirm whether the beam was really divided (Figure S2A, Supporting Information). A fiber‐coupled laser diode (LP852‐SF30, THORLABS, NJ, USA; wavelength = 852 nm) was used as the light source. The fiber‐coupled laser diode was controlled by a driver (CLD1010LP, THORLABS, NJ, USA).

### OCT Image Processing and 3D Rendering

The background signal measured when there was no sample was subtracted from all raw interference signals. Subsequently, the background‐subtracted interference signal was calibrated to correct the nonlinearity of the detected spectrum using linear interpolation. The calibrated interference signal was processed using a fast Fourier transform to generate a depth‐resolved backscattering profile. All OCT images were processed using C++ based algorithm and ImageJ (version 1.52p, US National Institutes of Health, MD, USA), and it represented pseudo‐color images. Cross‐sectional OCT images in two orthogonal planes were shown: transverse and coronal (Figure 1A). Coronal plane images were generated by re‐slicing transverse plane images using ImageJ. The 3D rendering was performed using a 3D viewer plugin of ImageJ. The transverse plane images of 640 frames were used to reconstruct a 3D rendering image. The epineurium was manually removed from each transverse plane image to visualize the individual myelinated axons under the epineurium. For a color‐coded 3D image, the blood vessel and Ni‐Ti wire were segmented manually in the transverse plane images.

### PSF Measurement and Analysis

The 3D PSF was measured to calculate the transverse resolution and extension of DOF (Figure S2B, Supporting Information). The 3D PSF was magnified using a 60× finite objective lens (33440, Edmund optics, NJ, USA) and imaged using the beam profiler. The transverse PSF was obtained by interpolating the x‐direction profiles of the radial beam distribution acquired at 10 µm increments in the axial direction using MATLAB 2019a. The transverse resolution was measured from the 3D PSF. Gaussian fitting was performed using MATLAB R2019a on the radial profiles of the x‐direction for each axial position. Transverse resolutions were determined as the FWHM of the Gaussian fitted data. Because astigmatism was not observed, the transverse resolution was calculated only on the radial profile of the x‐direction. The DOF was defined as an axial range where the transverse resolution was lower than 5 µm. In the general Gaussian beam distribution, twice the beam waist was equal to 1.7 times the FWHM of the beam distribution. According to the definition of the DOF, the DOF of the Gaussian beam with a waist of 1.0795 was 46.691 µm, which was 5.1 times shorter than the extended DOF of the dual‐focusing generator. All PSF analyses were performed with MATLAB R2019a using the measured 3D PSF. The 3D PSF was reconstructed using the 3D viewer plugin of ImageJ.

### Simulation for Multiple Scatterer

The x‐y plane (coronal plane) images blurred by two‐dimensional (2D) PSF were simulated according to the distance between two scatterers. The sizes of the two scatterers were the same. The diameter of a single scatterer was defined as 0.3 µm which was the same as the size of the bead for bead imaging. Distance between two scatterers were 0, 1.0, 1.5 2.0, 5.0 µm. Pixel resolution was 0.1 µm pixel^−1^. 2D PSFs were defined as the x‐y plane images of 3D PSF corresponding to C) to E) in Figure S3, Supporting Information. For considering a confocal effect, a product of the same 2D PSF was used in the simulation. The blurred images were simulated by convolution of scatterers' image with the product. All simulation was performed with MATLAB R2019a.

### Microbead Imaging

The microbeads were imaged using the imaging platform to verify the transverse resolution and extend the DOF of the dual‐focusing technique. The microbeads (LB3, Sigma Aldrich, MO, USA) with a diameter of 0.3 µm were diluted 10^5^ times in saline to ensure their proper distribution for rat sciatic nerve imaging setup. The microbeads (LB11, Sigma Aldrich, MO, USA) with a diameter of 1.1 µm were diluted 10^4^ times in saline to ensure their proper distribution for rabbit sciatic nerve imaging setup. The transverse and axial resolutions were calculated from the linear intensity profiles of the *x*– and *z*–axis, respectively. Gaussian fittings were performed on the intensity profiles of the main lobe using MATLAB R2019a. The transverse and axial resolutions were determined as the FWHM of the Gaussian fitted data. The intensities were determined as the logarithmic value of the peak intensity.

### Rat Model of WD

The right sciatic nerve of a Sprague Dawley (SD) rat (12 weeks old, Orient Bio Inc., Gyeonggi, Republic of Korea) was transected using micro scissors to induce WD. Subsequently, the epineurium was repaired using a 10‐0 suture (Polyamide monofilament, nonabsorbable surgical suture, Dafilon Black, Braun Korea Co., Ltd, Seoul, Republic of Korea). The surgical site was gently closed using a 5‐0 suture (nonabsorbable black silk 5‐0; Ailee Co., Ltd., Busan, Republic of Korea). During the entire surgical process, the rat was anesthetized with isoflurane (Hana Pharm Co., Ltd, Gyeonggi, Republic of Korea). The anesthesia progress was divided into two steps: induction and maintenance. Anesthesia was induced using 5% isoflurane mixed with oxygen, which was released at a rate of 1 L min^−1^ in an induction chamber. Subsequently, anesthesia was maintained with isoflurane at a concentration of 2.5–3.5%. The oxygen release rate was set at 1 L min^−1^. Cefazolin (200 mg kg^−1^, Cefazol, Eagle Vet. Tech Co., Ltd, Seoul, Republic of Korea) and ketoprofen (100 mg kg^−1^, UNI‐Ketopro, Uni Biotech Co., Ltd., Gyeonggi, Republic of Korea) were injected subcutaneously. The rat was returned to its cage and monitored every day. 7 days after surgery, WD was observed using the peripheral nerve imaging platform in vivo.

### Statistical Analysis of Axons

Statistical analysis was performed to compare the widths of linear structures of the normal myelinated axon and myelinated axon with WD quantitatively.

The widths of the linear structures were measured on profiles of the linear structures extracted from coronal plane OCT images (Figure S7A, Supporting Information). To extract the profiles of the linear structures, some post‐processing was performed. The logarithmic‐scaled OCT image was median filtered using a 3 × 3 window (Figure S7B, Supporting Information) and a region of interest (ROI) with size of 11 × 11 pixels was cropped (Figure S7C, Supporting Information). And then, ROI was binarized (Figure S7D, Supporting Information) using Otsu's method. And its orientation was calculated. The same ROI was also cropped and tilted from original OCT image (Figure S7E, Supporting Information) and was converted to linear scaled image (Figure S7F, Supporting Information). Finally, the vertical profile of the ROI was extracted from the center of the tilted OCT image. Gaussian fitting was performed on the vertical intensity profiles using MATLAB R2019a, and width with the 1/e^2^ values of maximum of the Gaussian fitted data were defined as the widths of the linear structures (Figure S7G, Supporting Information). In the coronal plane OCT images of the normal myelinated axon and myelinated axon with WD, the widths were randomly measured at 100 different positions for statistical analysis. The mean and standard deviation of the measured data were calculated and compared. An unpaired *t*‐test was performed to verify the difference in the width between the normal myelinated axon and myelinated axon with WD statistically. A histogram was created to compare the distributions of the width of the normal myelinated axon and myelinated axon with WD. All statistical analyses were performed using Prism (GraphPad Software, version 5.0, CA, USA).

### Rat Model with the Ni‐Ti Wire Inserted

The right sciatic nerve of the SD rat (12 weeks old) was pierced using the round needle connected to the Ni‐Ti wire (DYNALLOY, Inc., CA, USA). A round needle was used when inserting the Ni‐Ti wire because the rat sciatic nerve has a tough epineurium (Figure 5A). The diameter of the Ni‐Ti wire and width of the round needle were 0.05 and 0.56 mm, respectively. During the entire surgical process, the rat was anesthetized with isoflurane (Hana Pharm Co., Ltd, Gyeonggi, Republic of Korea). The process of inducing anesthesia was the same as that used for inducing WD.

### In Vivo Rat Sciatic Nerve Imaging for Myelinated Axons

One SD rat (13 weeks old) having health nerve and one SD rat (13 weeks old) with WD were imaged for normal myelinated axons and axons with WD, respectively. The rats were anesthetized with isoflurane (Hana Pharm Co., Ltd, Gyeonggi, Republic of Korea), shaved, and then, a muscle‐splitting incision was gently performed to expose the right sciatic nerve. The WD imaging site was 1.5 mm away from the transection site in the distal direction. Normal myelinated axons were also imaged at a similar position to that of WD. Before imaging, the sciatic nerve was fastened and lifted using a custom‐built nerve holder. Imaging optics were positioned near the region of interest using a two‐axis translation stage. To place the nerve within the imaging range, the distance between the nerve and imaging lens and the tilting angle of the nerve were adjusted precisely using a *z*–axis translational stage and a nerve holder, respectively. OCT imaging was performed at an A‐line rate of 250 kHz. The scanning speed of the fast axis was 250 Hz, and a slow scan was performed at 1.43‐µm interval. The laser power on the nerve surface was 20.7 mW, which was measured using a power meter (PM100D, THORLABS, NJ, USA) and a photodiode power sensor (S132C, THORLABS, NJ, USA).

The Institutional Animal Care and Use Committee of the Korea Institute of Science and Technology approved all the animal experiments (KIST‐2020‐048). All procedures involving the animals were performed according to the relevant guidelines and regulations.

### In Vivo Rat Sciatic Nerve Imaging for Connective Tissues, Blood Vessels, and Nerves with Ni‐Ti Wire Inserted

In vivo sciatic nerve imaging was performed to demonstrate the performance of the peripheral nerve imaging platform. One normal SD rat (12 weeks old) was used to image the epineurium and perineurium in vivo. Three normal SD rats (12 weeks old) were used for imaging adipose tissue, blood vessels, and sciatic nerve with Ni‐Ti wire inserted in vivo. The anesthetization method, surgical process, positioning process, A‐line rate, scanning speed, and laser power were the same as those described in previous section.

### Rat Sciatic Nerve Imaging with Ni‐Ti Wire Inserted Immediately after Euthanasia

The sciatic nerve of the SD rat (12 weeks old) with the NiTi wire inserted was imaged immediately after euthanasia using the imaging platform. One normal SD rat was used for imaging the sciatic nerve with a Ni‐Ti wire inserted immediately after euthanasia. The anesthetization method, surgical process, positioning process, A‐line rate, scanning speed, and laser power were the same as described previously. The Ni‐Ti wire was inserted into the right sciatic nerve. After the surgical procedure, the rat was euthanized in a carbon dioxide (CO_2_) chamber. The air in the chamber was replaced with CO_2_ at a rate of 10–30% per minute to induce analgesia. The CO_2_ exposure was maintained for more than 5 min.

### In Vivo Rabbit Sciatic Nerve Imaging

In vivo rabbit sciatic nerve imaging was performed to demonstrate the feasibility of applying the imaging platform to large animals for preclinical studies. One New Zealand White rabbit (1.5 kg, YONG BIO, Seoul, Republic of Korea) was used for in vivo sciatic nerve imaging. The rabbit was anesthetized with 3:1 tiletamine/zolazepam (11.5 mg kg^−1^, Zoletil, Virbac Korea, Seoul, Republic of Korea) and xylazine (Rompun, Bayer Korea, Seoul, Republic of Korea) mixture. During the experiment, an inhalation anesthetic (3% isoflurane) was used to maintain anesthesia. The left gluteal hind leg was shaved, and a muscle‐splitting incision was gently performed to expose the left sciatic nerve. The positioning process was the same as that described previously for in vivo rat sciatic nerve imaging. The A‐line rate, transverse scanning speed, and laser power were 50 kHz, 50 Hz, and 9.4 mW, respectively.

## Conflict of Interest

The authors declare no conflict of interest.

## Supporting information

Supporting Information

Supplemental Video 1

Supplemental Video 2

Supplemental Video 3

Supplemental Video 4

Supplemental Video 5

Supplemental Video 6

## Data Availability

The main data to support the findings of this study are available in articles and supplementary materials. Raw data and protocols can be provided from the corresponding authors upon reasonable request.
