# Supplementary material for: In Vivo Cellular‐Level 3D Imaging of Peripheral Nerves Using a Dual‐Focusing Technique for Intra‐Neural Interface Implantation
Source: Adv Sci (Weinh). 2021 Nov 29;9(3):2102876. doi: 10.1002/advs.202102876 (PMC8787432; doi:10.1002/advs.202102876)
Supplement: Supplementary file 1 — Supporting Information [file ADVS-9-2102876-s005.pdf]

WILEY-VCH

ADVANCED SCIENCE
Open Access

Supporting Information

for *Adv. Sci.*, DOI: 10.1002/advs.202102876

In Vivo Cellular-Level 3D Imaging of Peripheral Nerves Using a Dual-Focusing Technique for Intra-Neural Interface Implantation

*Min Woo Lee, Namseon Jang, Nara Choi, Sungwook Yang, Jinwoo Jeong, Hyeong Soo Nam, Sang-Rok Oh, Keehoon Kim, and Donghyun Hwang**

Supporting Information

In Vivo Cellular-Level 3D Imaging of Peripheral Nerves Using a Dual-Focusing Technique for Intra-Neural Interface Implantation

*Min Woo Lee, Namseon Jang, Nara Choi, Sungwook Yang, Jinwoo Jeong, Hyeong Soo Nam, Sang-Rok Oh, Keehoon Kim, and Donghyun Hwang**

Motion artifact suppression in rat sciatic nerve in vivo imaging

The A-line rate of 250 kHz was adopted during the in vivo imaging of the rat sciatic nerve to avoid the motion artifact caused by breathing. When the A-line rate was 50 kHz, i.e., one-fifth of 250 kHz, the motion artifacts were observed thrice in the coronal plane (Figure. 1A) image (red arrow heads in Figure S6A). The motion artifacts distorted the image severely and made it difficult to comprehend the nerve structure. The image distortion caused by motion artifacts can be viewed in the 3D volume rendering images (Figure S6B). Few motion artifacts were observed in both the coronal plane and the 3D rendering images when the A-line rate was 250 kHz (Figure S6C and D). For a high A-line rate, we acquired a motion artifact-suppressed volumetric image ($1.00 \times 1.00 \times 0.24$ mm^3) of the nerve microstructures in about 2.6 s. To the best of our knowledge, this imaging speed for volume is the highest among imaging devices that can show myelinated axons.

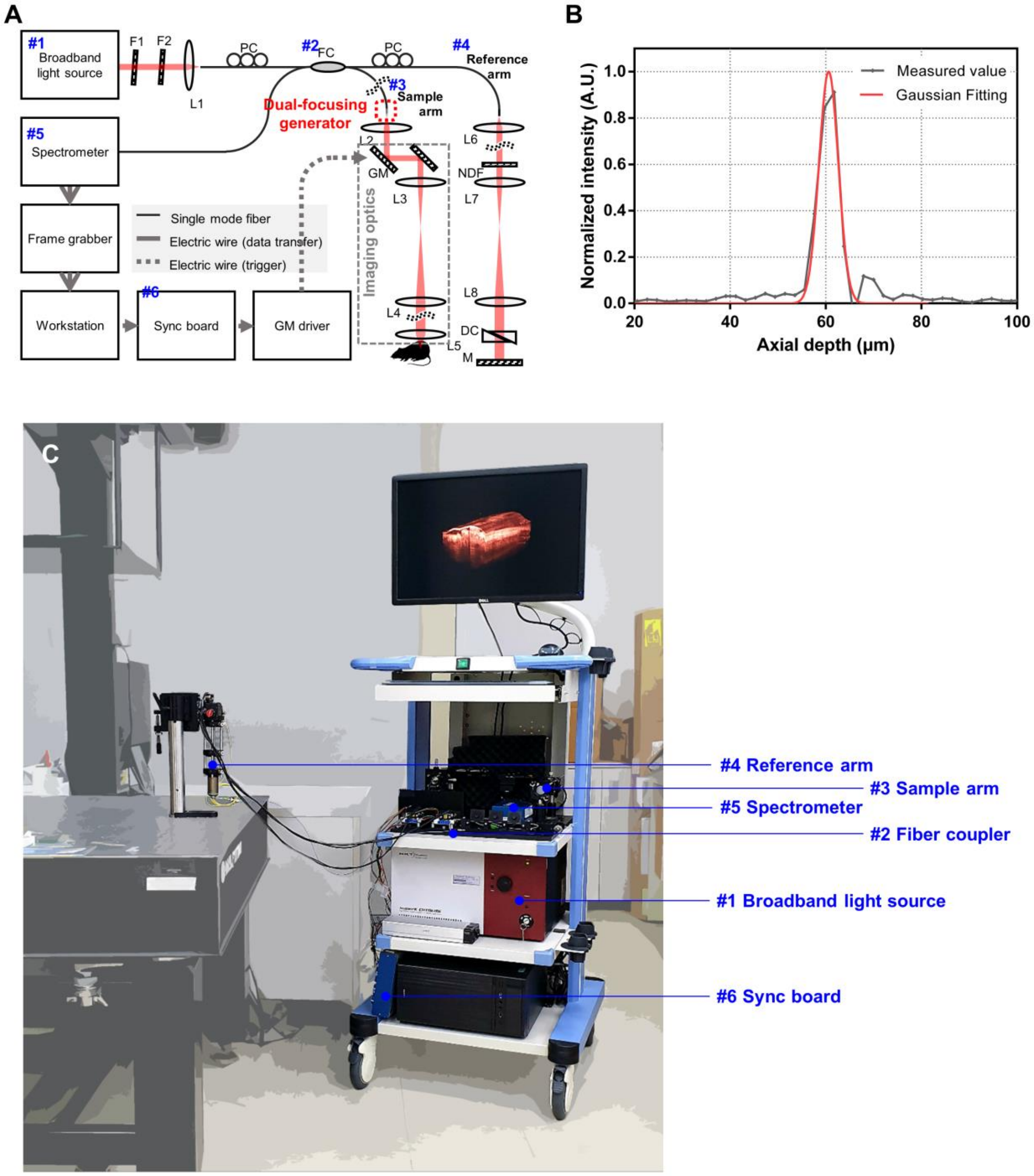

Figure S1 Optical coherence tomography-based peripheral nerve imaging platform for visualizing the microstructure of the peripheral nerve. A) Detailed schematic of the peripheral nerve imaging platform. F, filter; L, lens; PC, polarization controller; FC, fiber-optic coupler; GM, galvanometer mirror; NDF, neutral density filter; DC, dispersion compensator; and M, mirror. Black lines: single-mode fiber; Gray solid lines: electrical wire for transferring data; Gray dashed lines: electrical wire for triggering. B) Measured and Gaussian fitted data of the axial profile of the mirror C) Photograph of peripheral nerve imaging platform.

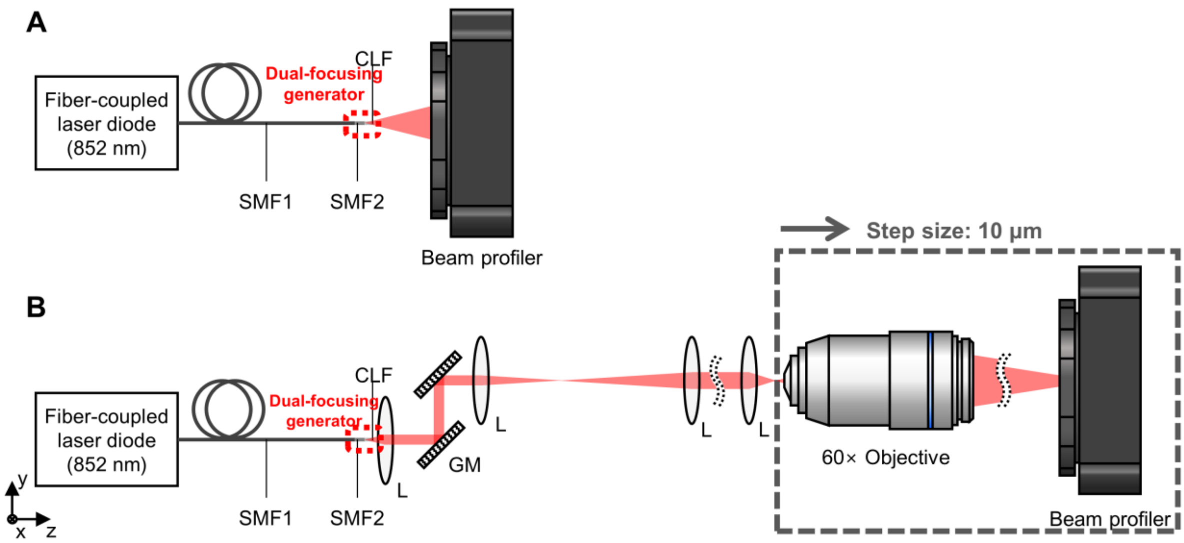

Figure S2 Experimental setup for measuring the output beam of the dual-focusing generator and three-dimensional (3D) point spread function (PSF). A) Experimental setup for measuring the output beam of the dual-focusing generator. SMF, single-mode fiber; CLF, coreless fiber. B) Experimental setup for measuring 3D PSF. L, lens and GM, galvanometer mirror.

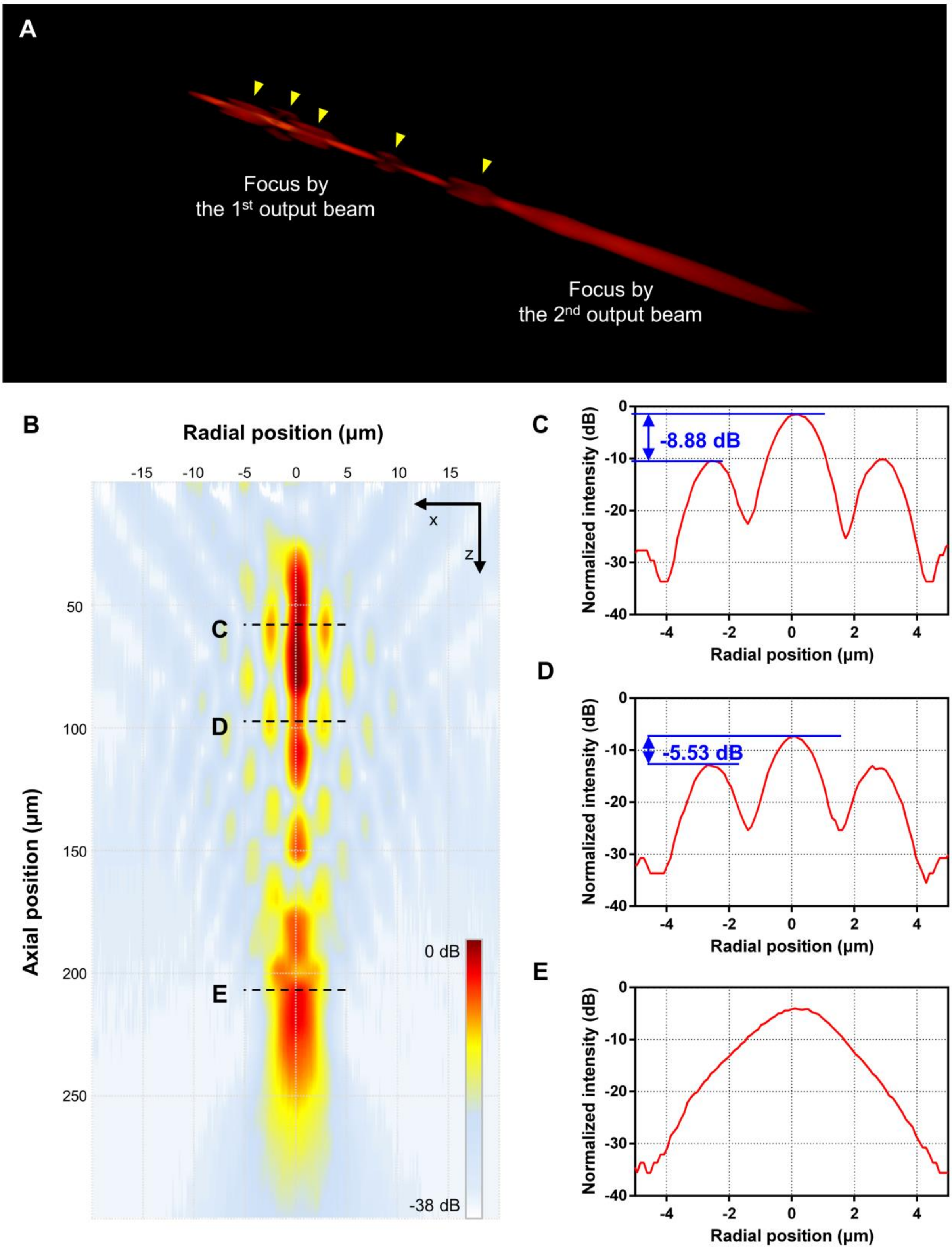

Figure S3 PSF analysis. A) Measured 3D PSF. Since the first output beam has a ring shape, the Bessel beam features such as the side lobe (yellow arrow heads) are shown in the PSF. B) Square of the transverse PSF corresponding to Figure. 1E, which is proportional to the detected signal due to the confocal gating effect. C-E) Normalized radial intensity profiles at four different depth positions indicated in B. Since the minimum difference between the main

and side lobes is around −5.94 dB, a little side lobe artifact can be shown in the OCT images. In the radial profile of focus by the second output beam, the side lobe is not demonstrated because of the Gaussian beam property

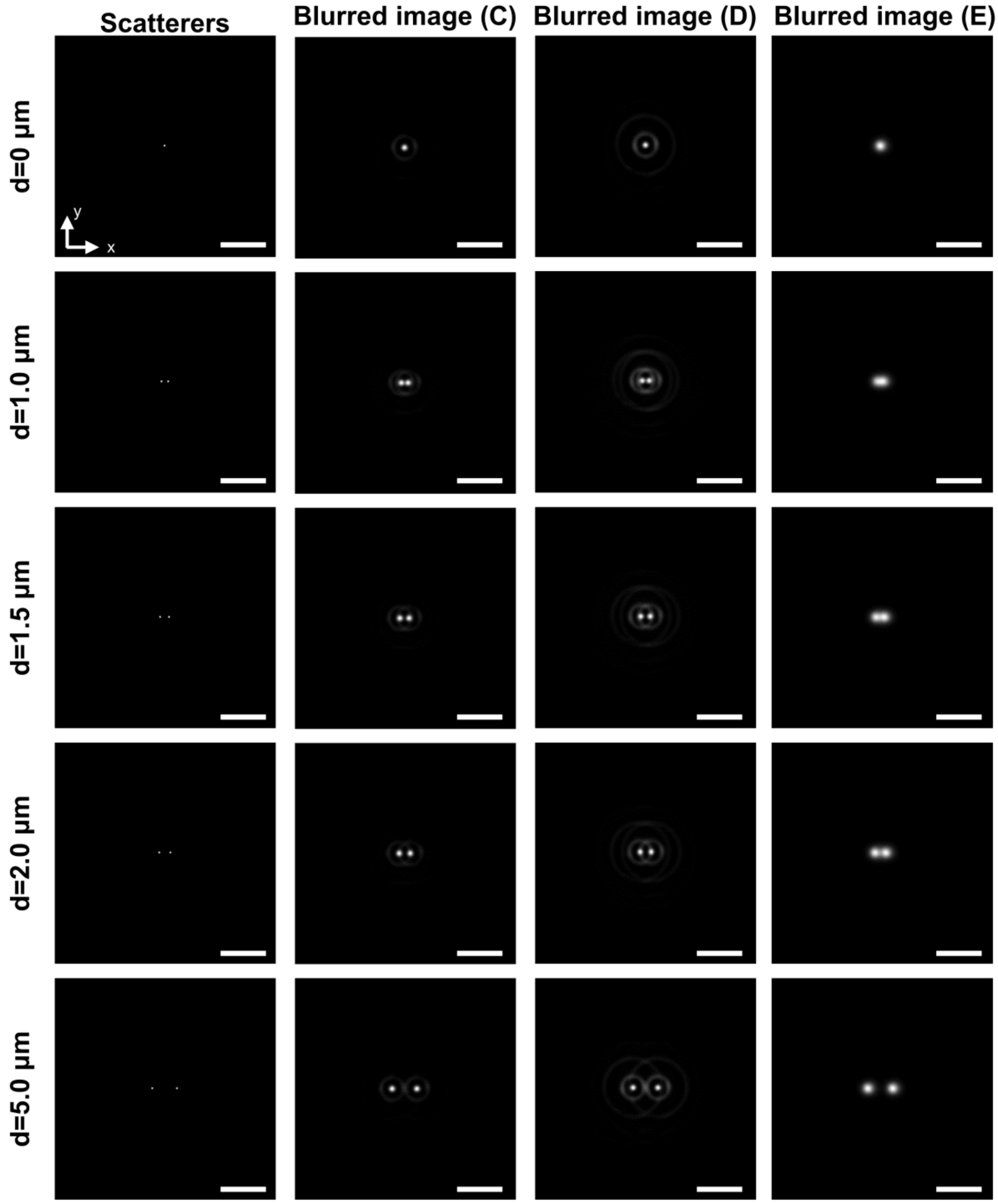

Figure S4 Simulated results for multiple scattering. Each row shows the simulated images corresponding to each distance between scatterers. The first column shows THE simulated image before being blurred by PSF. The second through fourth columns shows blurred images by two-dimensional (2D) PSF corresponding to C to D in Figure S3. When the distance between scatterers is greater than 1.5 μm, two scatterers were clearly differentiated in spite of being blurred by 2D PSF of C and D. Although it is only a narrow area of the entire PSF, in the blurred image by the 2D PSF of D, considerable modulation by the side lobes, an inherent limitation of the DOF extending technique using the Bessel beam, was observed. On the other

hand, in the blurred images by 2D PSF of E having Gaussian beam property, any modulation was not observed. d, the distance between scatterers All scale bars: 100 μm.

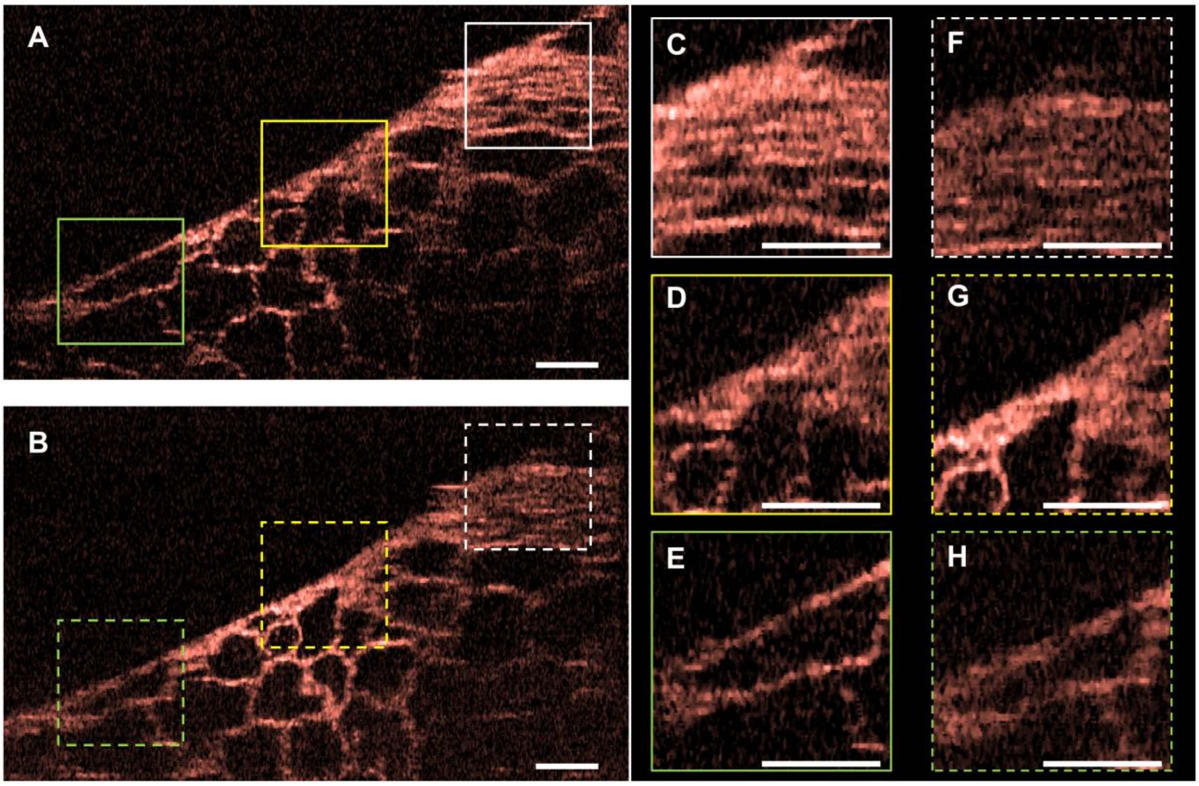

Figure S5 Validation of depth of focus (DOF) extending performance by imaging a biological sample. Representative cross-sectional OCT images of a fresh grape A) with and B) without the dual-focusing generator. C-E) Magnified images corresponding to the rectangular region labeled in A). F-H) Magnified images corresponding to the rectangular region labeled in B). When the dual-focusing generator was applied, although a slight decrease in sensitivity was observed (D and G), the cellular wall structures of the grape were visualized with high resolution throughout the extended focal range. On the other hand, When the dual-focusing generator was not applied, the wall structures of the grape are clearly visualized only in the focal region (G). In the out of focus region (F and H), cellular wall structures of the grape are blurred. All scale bars: 100 μm.

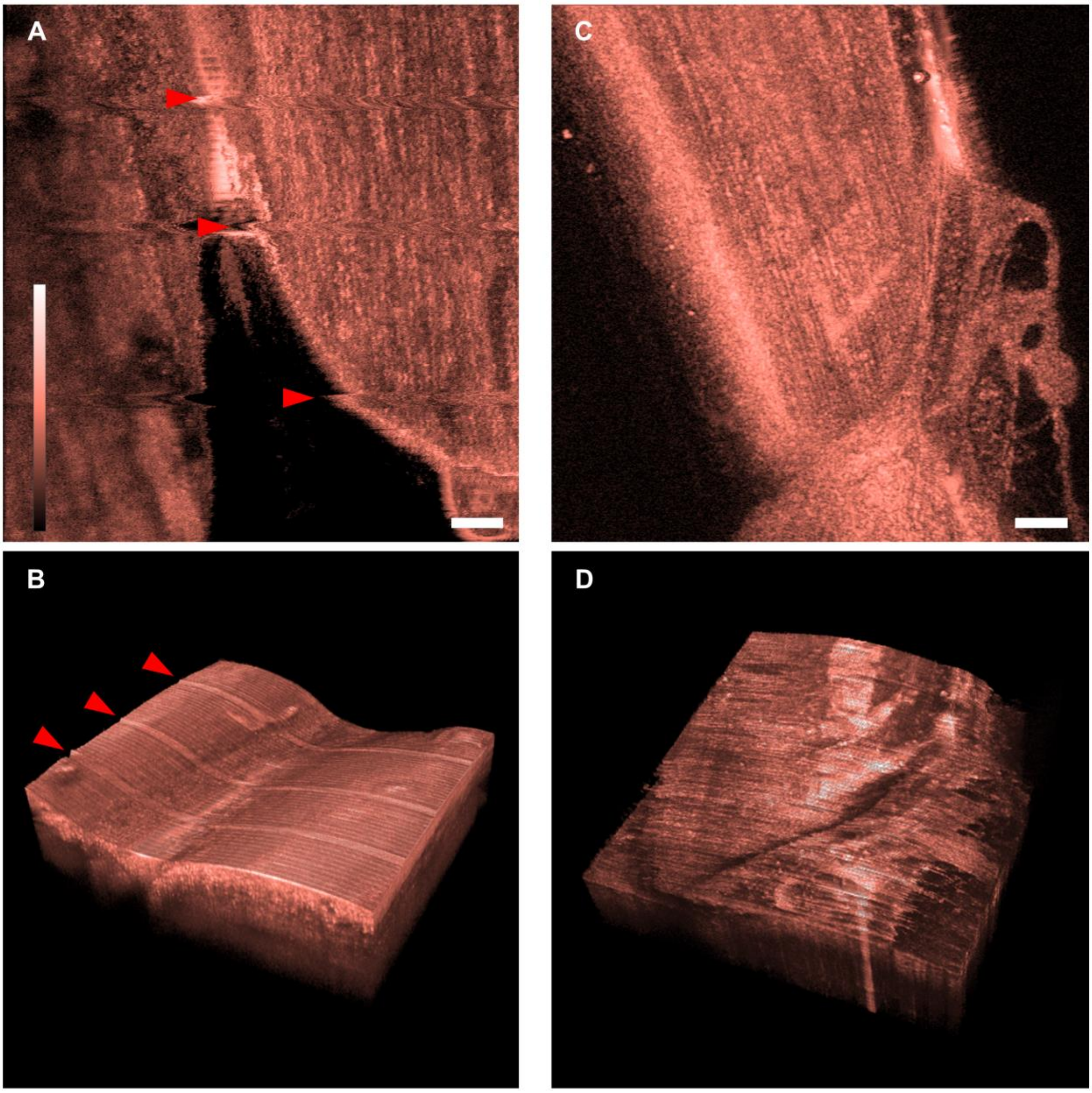

Figure S6 Motion artifact-suppression performance of imaging platform. A) Coronal plane image and B) corresponding 3D volume rendering image of the rat sciatic nerve when the A-line rate is 50 kHz. Red arrowheads indicate motion artifacts caused by breathing. Severe image distortion makes it difficult to observe the structure of the myelinated axons. C) Coronal plane image and D) corresponding 3D volume rendering the image of the rat sciatic nerve when the A-line rate is 250 kHz. Without the motion artifact, the line structures of the myelinated axons were observed clearly. Scale bar: 100 µm.

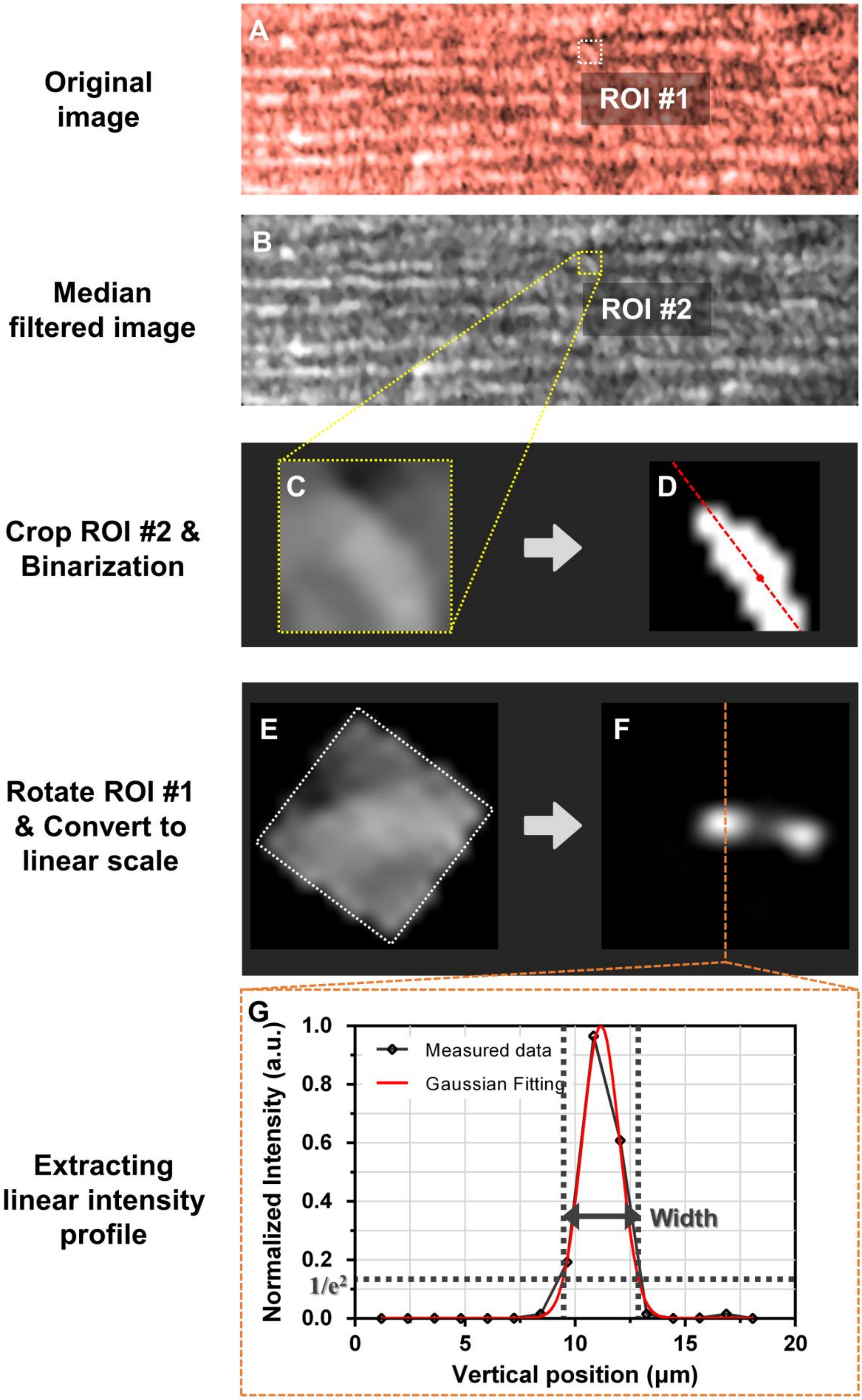
Original image
A
ROI #1
Median filtered image
B
ROI #2
Crop ROI #2 & Binarization
C
D
Rotate ROI #1 & Convert to linear scale
E
F
Extracting linear intensity profile
G
Measured data
Gaussian Fitting
Width
1/e²
Normalized Intensity (a.u.)
Vertical position (μm)
0.0
0.2
0.4
0.6
0.8
1.0
0
5
10
15
20

Figure S7 Extracting process for a linear scaled profile of myelinated axon. A) Logarithmic scaled original OCT image of myelinated axons and selection of region of interest (ROI) #1 (white dashed box). B) Median filtered image and selection of ROI #2 (yellow dashed box). C) Cropped ROI #2. D) Binarized ROI, its centroid (red dot) and orientation (red dashed line). E) Cropped and rotated ROI #1 based on centroid and orientation calculated in D). F) linear scaled ROI converted from E). G) linear intensity profile of the individual myelinated axon corresponding to orange dashed line in F).
